# Supplementary material for: Novel Driver Strength Index highlights important cancer genes in TCGA PanCanAtlas patients
Source: PeerJ. 2022 Aug 11;10:e13860. doi: 10.7717/peerj.13860 (PMC9375969; doi:10.7717/peerj.13860)
Supplement: Supplemental Information 2 [file peerj-10-13860-s002.zip › COHORTS/genes plots/detailed/2021_8_16_14_9_distribution_events_detailed_18.pdf]

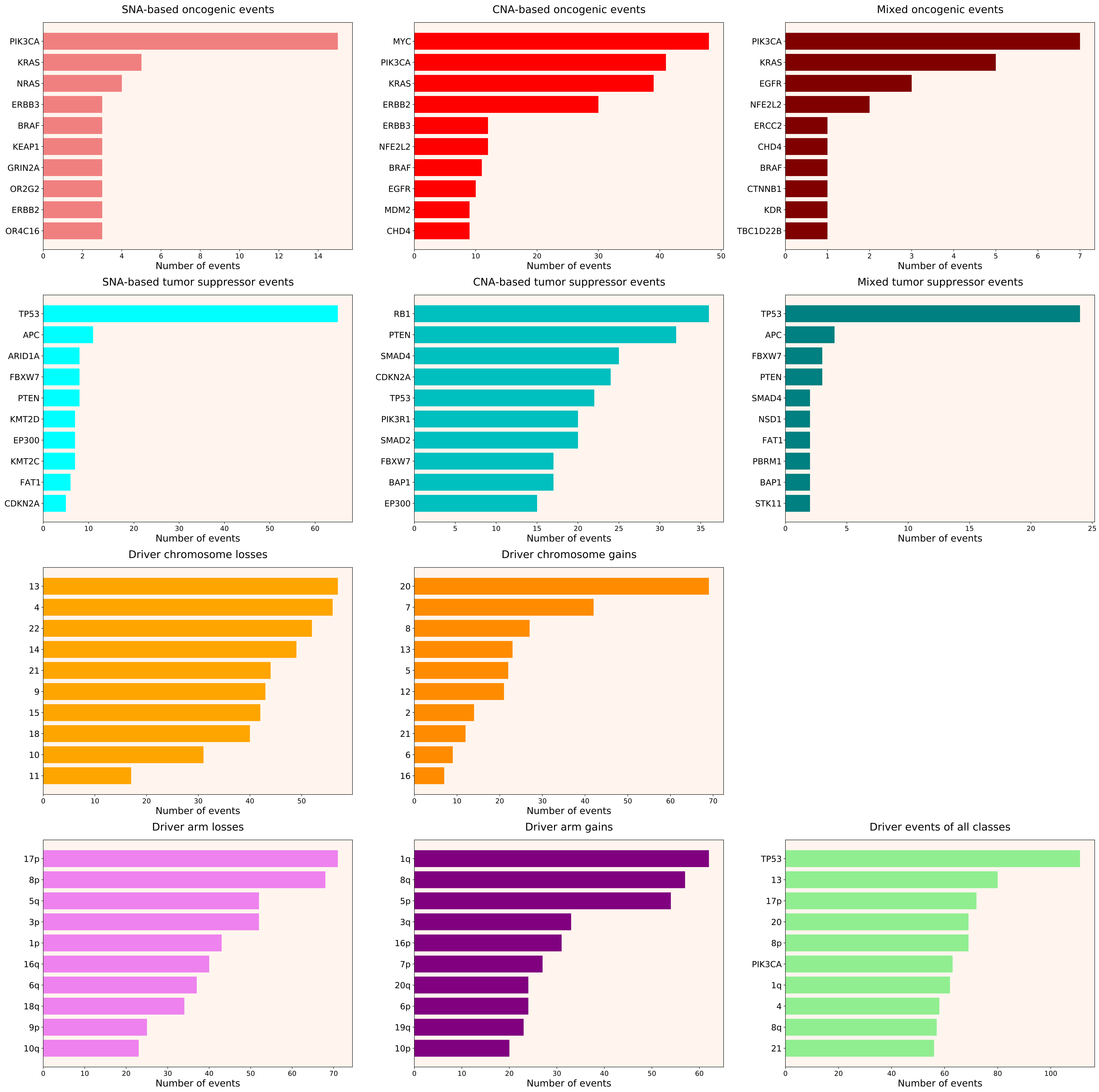
SNA-based oncogenic events
PIK3CA
KRAS
NRAS
ERBB3
BRAF
KEAP1
GRIN2A
OR2G2
ERBB2
OR4C16
0 2 4 6 8 10 12 14
Number of events
CNA-based oncogenic events
MYC
PIK3CA
KRAS
ERBB2
ERBB3
NFE2L2
BRAF
EGFR
MDM2
CHD4
0 10 20 30 40 50
Number of events
Mixed oncogenic events
PIK3CA
KRAS
EGFR
NFE2L2
ERCC2
CHD4
BRAF
CTNNB1
KDR
TBC1D22B
0 1 2 3 4 5 6 7
Number of events
SNA-based tumor suppressor events
TP53
APC
ARID1A
FBXW7
PTEN
KMT2D
EP300
KMT2C
FAT1
CDKN2A
0 10 20 30 40 50 60
Number of events
CNA-based tumor suppressor events
RB1
PTEN
SMAD4
CDKN2A
TP53
PIK3R1
SMAD2
FBXW7
BAP1
EP300
0 5 10 15 20 25 30 35
Number of events
Mixed tumor suppressor events
TP53
APC
FBXW7
PTEN
SMAD4
NSD1
FAT1
PBRM1
BAP1
STK11
0 5 10 15 20 25
Number of events
Driver chromosome losses
13
4
22
14
21
9
15
18
10
11
0 10 20 30 40 50
Number of events
Driver chromosome gains
20
7
8
13
5
12
2
21
6
16
0 10 20 30 40 50 60 70
Number of events
Driver arm losses
17p
8p
5q
3p
1p
16q
6q
18q
9p
10q
0 10 20 30 40 50 60 70
Number of events
Driver arm gains
1q
8q
5p
3q
16p
7p
20q
6p
19q
10p
0 10 20 30 40 50 60
Number of events
Driver events of all classes
TP53
13
17p
20
8p
PIK3CA
1q
4
8q
21
0 20 40 60 80 100
Number of events
